# Forearm rotation improves after corrective osteotomy in patients with symptomatic distal radius malunion

**DOI:** 10.1016/j.heliyon.2024.e29570

**Published:** 2024-04-16

**Authors:** E.M. van Es, M. Dijkhof, J.S. Souer, F.J. van Ewijk, L. Hoogendam, H.P. Slijper, R.W. Selles, J.W. Colaris, Hand-Wrist Study Group, R.A.M. Blomme, J.M. Smit, K. Harmsen, G. Halbesma, G.M. Vermeulen, JP de Schipper, J.H. van Uchelen, O.T. Zöphel, J.S. Souer, L. Esteban Lopez, A. Fink, R. van Huis, P.Y. Pennehouat, K. Schoneveld, G.D. Arends, R. Feitz, L. Hoogendam, S.E.R. Hovius, Y.E. van Kooij, J.E. Koopman, M.J.W. van der Oest, W.A. de Ridder, R.W. Selles, L. Sikking, H.P. Slijper, M.H.P. ter Stege, J.S. Teunissen, R.M. Wouters, N.L. Loos, N.H.A. Mendelaar, L van Wijk, W.R. Bijlsma, L.S. Duraku, E.P.A. van der Heijden, C.A. Hundepool, J.M. Zuidam

**Affiliations:** a Department of Orthopaedics and Sports Medicine, Erasmus University Medical Center, Rotterdam, the Netherlands; b Hand and Wrist Center, Xpert Clinics, Amsterdam, the Netherlands; c Center for Hand Therapy, Xpert Clinics, Utrecht, the Netherlands; d Department of Plastic, Reconstructive and Hand Surgery, Erasmus University Medical Center, Rotterdam, the Netherlands; e Department of Rehabilitation Medicine, Erasmus University Medical Center, Rotterdam, the Netherlands; f Department of Orthopaedics and Sports Medicine, Erasmus University Medical Center, Rotterdam, the Netherlands

**Keywords:** Distal radius malunion, Forearm rotation, Range of motion, Patient-reported outcome measurements

## Abstract

**Objectives:**

Distal radius malunion can result in pain and functional complaints. One of the functional problems that can affect daily life is impaired forearm rotation. The primary aim of this study was to investigate the effect of corrective osteotomy for distal radius malunion on forearm rotation at 12 months after surgery. We secondarily studied the effect on grip strength, radiological measurements, and patient-reported outcome measurements (PROMs).

**Patients and methods:**

This cohort study analysed prospectively collected data of adult patients with symptomatic distal radius malunion. All patients underwent corrective osteotomy for malunion and were followed for 1 year. We measured forearm rotation (pronation and supination) and grip strength and analysed radiographs. PROMs consisted of the Patient-Rated Hand/Wrist Evaluation (PRWHE) questionnaire, Visual Analogue Scale for pain, and satisfaction with hand function.

**Results:**

Preoperative total forearm rotation was 112° (SD: 34°), of which supination of 49° (SD: 25°) was more impaired than pronation of 63° (SD: 17°). Twelve months after surgery, an unpaired Student's *t*-test showed a significant improvement of total forearm rotation to 142° (SD: 17°) (p < 0.05). Pronation improved to 72° (SD: 10°), and supination to 69° (SD: 13°) (p < 0.05). Grip strength, PROMs, as well as inclination and volar tilt on radiographs improved significantly during the first year after surgery (p < 0.05).

**Conclusion:**

In patients with reduced forearm rotation due to distal radius malunion, corrective osteotomy is an effective treatment that significantly improves forearm rotation. In addition, this intervention improves grip strength, the PRWHE-score, pain, and satisfaction with hand function.

## Highlights

• Distal radius malunion can result in symptomatic limitation in forearm rotation, affecting patients' daily activities.
• Forearm rotation is an essential function, especially in today's digital age where activities like using a mobile phone or keyboard are important.
• In patients with reduced forearm rotation due to distal radius malunion, corrective osteotomy is an effective treatment to improve this function.

## 1 Introduction

Distal radius fractures are the most common fractures of the upper extremity seen in the emergency department. In The Netherlands, the incidence of distal radius fractures is estimated at about 20 per 10,000 persons per year [1]. In particular, individuals aged >60 and women are at greater risk of fracturing their distal radius. Moreover, these patients more often receive conservative treatment, thereby increasing the incidence of malunion in this group [2]. A malunion of the distal radius is observed in 24% and 11% of conservatively treated patients and those treated operatively for a distal radius fracture, respectively [3].

The morphological characteristics of malunions can differ due to different types of fractures and displacement. There is a three-dimensional deformity involving translation, angular, and rotational deformity. This three-dimensional deformity can alter the congruence of the distal radioulnar joint (DRUJ) [4]. Although some patients remain asymptomatic, this change in anatomy may result in various symptoms, including stiffness, decreased range of motion (ROM), decreased grip strength, pain, and reduced cosmetic appearance [5]. Patients with a malunion who suffer from these symptoms may benefit from a corrective osteotomy [3]. The aim of this surgery is to improve the alignment of the distal radius to within acceptable parameters, including a better congruency of the DRUJ, in an attempt to improve function and reduce pain [6].

Most research conducted on corrective osteotomies of the distal radius has focused on the improvement of radiographic parameters, such as inclination angle, volar tilt, and length versus the ulna [2,3,6,7]. However, as clinical outcomes do not always correlate with radiologic results, it is important to also measure clinical parameters such as ROM, grip strength, and functional outcomes [8]. ROM, particularly forearm rotation (pronation and supination), is essential for daily functioning [9,10]. Forearm rotation has always been an important function (e.g., using a fork or opening a door). However, in today's digital age, even greater rotations are required in daily activities (e.g., using a keyboard or cell phone) [11]. The treatment of distal radius malunion with impaired forearm rotation is challenging [12].

To date, there are small cohort studies investigating the effect of corrective osteotomy for distal radius malunion on forearm rotation. Although some evidence suggests improvement in forearm rotation, those studies have small sample sizes (range: 11–37 patients) or follow-up periods of less than one year [[5], [6], [7],[13], [14], [15], [16]]. Therefore, the primary goal of this study is to analyse a large patient population and investigate the effect of corrective osteotomy for distal radius malunion on forearm rotation at 12 months after surgery. We hypothesised that the forearm rotation would improve significantly at 12 months after surgical correction of the distal radius malunion. The secondary goals of this study are to analyse the effect of a corrective osteotomy on grip strength, radiological measurements, and patient-reported outcome measurements (PROMs).

## 2 Methods

### 2.1 Study design

This cohort study retrospectively analysed prospectively collected data at the Xpert Clinics (Amsterdam, The Netherlands). This clinic is specialised in hand and wrist surgery. Currently, there are 25 locations, 23 European Board-certified (Federation of European Societies for Surgery of the Hand) hand surgeons, and >150 hand therapists are employed by these clinics. Routinely, outcome measures before and up to 1 year after treatment are collected for all patients. Before their first visit to the clinic, patients were invited to fill in a web-based intake questionnaire with questions about their general health and specific complaints of their hand and wrist. From the start of treatment Patient-Reported Outcome Measurements (PROMs) including pain, hand function, aesthetics, and satisfaction with hand/wrist function were taken at predefined timepoints using GemsTracker [17]. The Clinician-Reported Outcome Measurements include grip strength and ROM. Further information on the study cohort has been published previously [17]. The reporting of this study was conducted using the observational routinely-collected data statement (RECORD) [18]. The study was performed according to the principles of the Declaration of Helsinki and approved by the Medical Ethical Committee of the Erasmus Medical Center (reference number: MEC-2018-1088). All patients provided written informed consent for the use of their anonamyzed data.

### 2.2 Patients

All patients who visit our clinic with symptoms after a malunited distal radius fracture were evaluated by a hand surgeon. Conservative treatment options, including hand therapy, brace therapy or steroid injections were started first. Only patients with persistent complaints of pain and/or functional impairment were eligible for a corrective osteotomy. In this study, we included patients aged >18 who underwent corrective osteotomy for a symptomatic distal radius malunion between 2011 and 2019. We excluded patients with missing baseline or follow-up data regarding pronation and supination. Of them, only patients with 12-months follow-up data could be included in the primary analysis.

### 2.3 Treatment

At our clinic, we usually perform corrective osteotomies for distal radius malunion through a volar modified Henry approach. Volar tilt, radial inclination, radial length, and rotational malalignment are corrected at the same time if needed. The surgery was preoperatively planned based on the radiographs and using the contralateral side as a reference for the ulnar variance. The use of fluoroscopy during the procedure made it possible to adjust this. Rotational malalignment was evaluated and corrected if possible during surgery as this could not be diagnosed on conventional radiographs. The affected arm is anaesthetised with an axillary or supraclavicular brachial plexus block. The hand surgeon fixates the osteotomy distally first and uses a lamina spreader to gain length and correct alignment, followed by proximal fixation with a VariAx plate (Stryker, Kalamazoo, MI, USA). An additional dorsal approach can be performed when the correction is not sufficiently correctable through a volar approach only. Although using a bone graft is not our standard practice, this method can be used at the discretion of the treating surgeon only when the surgeon did not expect enough stability and bone healing without a graft. Perioperatively, forearm function is tested, and radiological control is performed using fluoroscopy. After closing the wound, a below-elbow plaster cast is applied.

During the first few weeks after surgery, all patients are treated by a hand therapist. Rehabilitation consisted of three phases. The first phase aims to stimulate finger, upper arm, and forearm ROM. After 5–7 days, the below-elbow cast is removed, a removable volar wrist splint is applied, and pronation and supination exercises are initiated. The second phase starts four weeks after surgery. In this phase, ROM and functionality are optimised, and usage of the wrist splint is reduced. The third phase starts approximately 12 weeks postoperatively. In this final phase, the aim is to improve functionality and fully reintegrate back into everyday life. Three months postoperatively, lateral and posteroanterior radiographs of the wrist are obtained, and the surgeon examines the patient. Minor differences in treatment and rehabilitation may occur because of the preferences of the surgeon or hand therapist and individual patient differences.

### 2.4 Measurements

The primary outcome of this study was forearm rotation at 12 months post-surgery versus pre-surgery (baseline). We also assessed forearm rotation at 3 months versus baseline and between 3 and 12 months. In addition, PROMs, grip strength, and radiographic measurements were collected preoperatively and at various time points postoperatively.

Baseline patient characteristics and treatment parameters were collected from the database and electronic patient files. Physical examination of the forearm was performed at baseline and 3 and 12 months post-surgery. As prescribed by the American Society of Hand Therapists, forearm rotation (sum of pronation and supination) was measured using a universal goniometer [19]. With this method, a good to excellent interclass (0.83–0.92) and intraclass (0.92–0.94) reliability was previously found [20]. We used an existing database of 65 patients undergoing a corrective osteotomy of the distal radius with complete ROM measurements prior to surgery and 12 months postoperatively to calculate a distribution-based minimal clinically important difference (MCID). We defined the MCID as 0.5 the standard deviation of preoperative forearm rotation [21]. This resulted in an MCID of 19° in forearm rotation for patients undergoing a corrective osteotomy of the distal radius.

Grip strength was measured using the E-link system (Biometrics Ltd, Newport, UK). Patients sat in a chair with the elbow in 90° flexion and the forearm in the neutral position. The mean grip strength (position two of the dynamometer) of three affected and non-affected wrist attempts was recorded [19].

PROMs were collected and managed using an electronic data capture tool at baseline and 6 weeks, and 3, 6, and 12 months post-surgery. We used the Dutch version of the Patient-Rated Wrist/Hand Evaluation (PRWHE) to measure pain and function. The PRWHE is a validated questionnaire for evaluating hand and wrist problems and consists of 15 questions: 5 for pain and 10 for function. The total PRWHE score ranges from 0 to 100 (0–50 for pain and 0–50 for function), with a score of 100 indicating the worst outcome [22]. Furthermore, the experience of pain in the previous week and satisfaction with hand/wrist function were evaluated using a 10 cm Visual Analogue Scale (VAS). The VAS score ranges from 0 to 100, with 100 representing the worst pain or best satisfaction score.

The radiographic evaluation consisted of standard posteroanterior and lateral radiographs obtained at baseline and 3 months post-surgery. We measured radial inclination (°), and ulnar variance (−/0/+) on the posteroanterior radiographs and volar tilt (°) on the lateral radiographs using the method described by Kreder et al. [23]. Because the radiographs were not calibrated, we used a classification of negative (−), neutral (0), and positive (+) ulnar variance based on visual assessment. Two authors (EE, MD) independently performed all radiographic measurements using the integrated software of the Picture Archiving and Communication System.

### 2.5 Statistical analysis

Patient characteristics and surgical details are shown as descriptive values. A p-value <0.05 denoted a statistically significant difference between the groups at baseline. Considering the large sample size and single population in this study, an unpaired Student's *t*-test was used to determine differences in forearm rotation, grip strength, PROMs, and radiograph measurements at baseline and 3 and 12 months after corrective osteotomy. The inter-observer reliability was evaluated using the intraclass correlation coefficient (ICC) for the radiographic measurements. We used a two-way mixed model with an absolute agreement and a confidence interval (CI) of 95%. Statistical analyses were performed using SPSS version 25.0 (IBM Corporation, Armonk, NY, USA).

## 3 Results

**Fig. 1:**
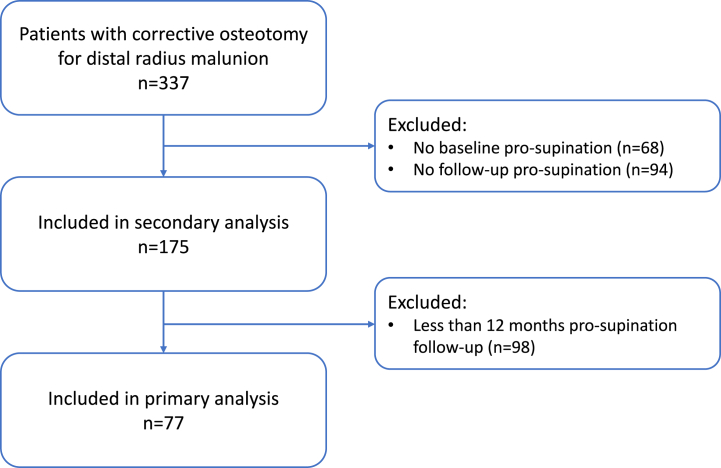
Inclusion flowchart of the study. Patients who were followed up for 12 months months in terms of pronation and supination were included in the primary analyses.

**Fig. 2:**
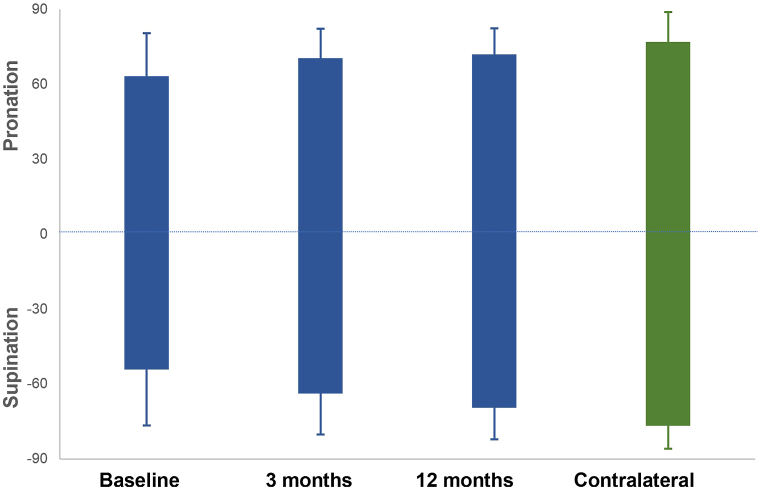
Forearm rotation at baseline and 3 and 12 months after surgery from the affected and contralateral sides. Boxes display the mean pronation (above the horizontal line) and supination (below the horizontal line). Whiskers display standard deviations.

Eight different hand surgeons performed a corrective osteotomy for distal radius malunion in 337 patients between 2011 and 2019. The total eligible group consisted of 175 patients, which we included in the secondary analysis. Of these patients, 77 had data obtained from baseline and at least 12-months follow-up goniometry measurement and could thererefore be included in the primary analysis. The characteristics of both the total group and the primary analysis group at baseline are shown in Table 1. Fig. 1 demonstrates the inclusion flowchart. The subgroup of patients included in the primary analysis had similar characteristics to the whole group. Most patients in this group were female with a mean age of 56 years and had been treated primarily mainly conservatively (see Table 2) (see Fig. 2).

**Table 1:** Baseline characteristics of the total study population, the 12-month follow-up group, and the <12-month follow-up group.

Characteristic	*Total study population n = 175*	12-month *follow-up group n* = *77*	*<*12-month *follow-up group n = 98*	p-value
*Sex (female)*	82	87	79	0.15
*Age (years)*	55 ± 14	56 ± 12	55 ± 15	0.57
*BMI (kg·m*^*−*^*^2^)*	25 ± 4	25 ± 4	25 ± 4	0.78
*Alcohol (yes)*	40	36	43	0.38
*Smoking (yes)*	19	25	15	0.12
*Workload*				0.67
*No work*	40	35	43	
*Light physical work*	25	27	24	
*Moderate physical work*	22	22	22	
*Heavy physical work*	13	16	11	
*ASA class*				0.05
*1*	73	65	79	
*2*	27	35	21	
*Surgery on the dominant hand (yes)*	47	48	46	0.78
*Ipsilateral wrist history (yes)*	31	38	26	0.08
*Time from trauma to surgery (months)*	17 ± 36	21 ± 49	13 ± 23	0.16
*Duration of symptoms (months)*	12 ± 19	13 ± 19	11 ± 19	0.06
*Prior treatment*				0.12
*Conservative*	87	83	90	
*K-wire fixation*	5	7	4	
*Plate fixation*	3	4	2	
*Corrective osteotomy*	3	4	2	
*External fixator*	2	2	2	

ASA class: American Society of Anesthesiologists Classification; BMI: body mass index; n: number of patients. Values are presented as the mean ± standard deviation or percentage (%).

**Table 2:** Forearm rotation at baseline, 3 and 12 months after surgery, and from the contralateral side for the total study population and the primary analysis group.

	Number of patients	Mean ± SD	
*Forearm rotation (°)*			
*Baseline*	175	117 ± 31	
3 months	159	134 ± 23	
12 months	77	142 ± 17	
*Contralateral*	129	154 ± 19	
*Pronation (°)*			
*Baseline*	175	63 ± 17	
12 months	78	72 ± 10	
*Contralateral*	129	77 ± 9	
*Supination (°)*			
*Baseline*	175	54 ± 22	
12 months	77	69 ± 13	
*Contralateral*	132	77 ± 12	
	**Mean difference ± SD**	**95% CI**	**p-value**
*Forearm rotation (°)*			
*Baseline vs* 3 months	17 ± 27	11–23	<0.001
*Baseline vs* 12 months	25 ± 35	18–32	<0.001
3 months *vs* 12 months	8 ± 16	3–13	0.008

CI: confidence interval; SD: standard deviation.

All patients underwent corrective osteotomy of the distal radius using the opening wedge technique, with a volar approach in 172 patients (98%) and a combined volar and dorsal approach in 3 patients (2%). In 103 of the 175 patients, only the volar tilt or the radial inclination was corrected. In the remaining 72 patients, the radius was also lengthened. No bone grafts were needed.

### 3.1 Forearm rotation

Before surgery, the mean forearm rotation in the primary analysis group was 112° (SD: 34°). At 12 months, it improved significantly to 142° (SD: 17°) (p < 0.05). This corresponded to 94% of the value obtained for the contralateral side. Most improvement in forearm rotation was seen in the first 3 months after surgery (increase of 21°; SD: 29°) (p < 0.05). Between 3 and 12 months, we recorded a significant improvement of another 9° (SD: 19°) (p < 0.05). When total forearm rotation is divided into pronation and supination, it appears that pronation improves significantly only in the first 3 months, while supination continues to improve between 3 and 12 months after surgery (p < 0.05).

**Fig. 3:**
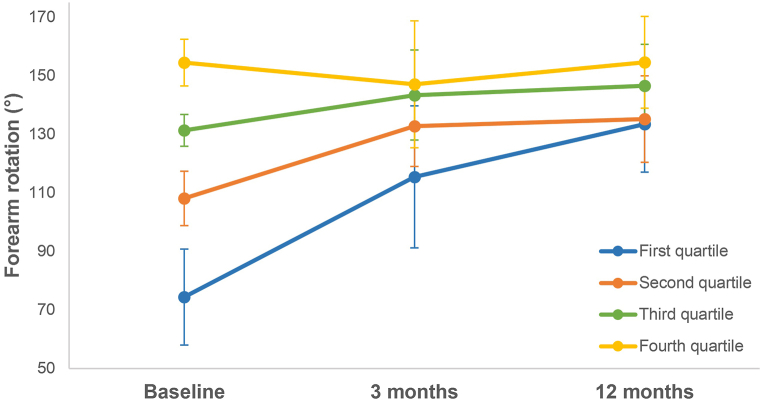
Forearm rotation is divided into quartiles. The first (blue) indicates the worst quartile, while the fourth (yellow) illustrates the best quartile of baseline rotation. Markers display mean forearm rotation; error bars display standard deviations.

At 3 months postoperatively, 46.2% of the patients achieved the MCID of 19° in forearm rotation; this proportion increased to 61.5% after 12 months. We subsequently classified patients into quartiles based on their baseline forearm rotation to obtain more insight into the course of the change in forearm rotation resulting from the corrective osteotomy. The first quartile represents patients with the worst baseline forearm rotation (range 31°–94°). Similarly, second (range 95°–122°), third (range 122°–142°), and fourth (range 142°–171°) quartile show patients with better and best forearm rotation at baseline. The mean improvement in forearm rotation was 59° (SD: 24°), 27° (SD: 19°), 15° (SD: 16°), and 0° (SD: 14°) from the first to fourth quartile, respectively. This improvement was significant in the first, second, and third quartiles (p < 0.05). At 12 months of follow-up, there were no significant differences between the groups (Fig. 3).

### 3.2 Grip strength

Grip strength increased significantly from 15 kg (SD: 10 kg) at baseline to 18 kg (SD: 8 kg) and 23 kg (SD: 8 kg) at 3 and 12 months postoperatively, respectively (p < 0.05). These values corresponded to 56%, 67%, and 85% of the values obtained for the contralateral side (27 kg; SD: 10 kg) in 156, 149, and 76 patients, respectively.

### 3.3 PROMs

**Fig. 4:**
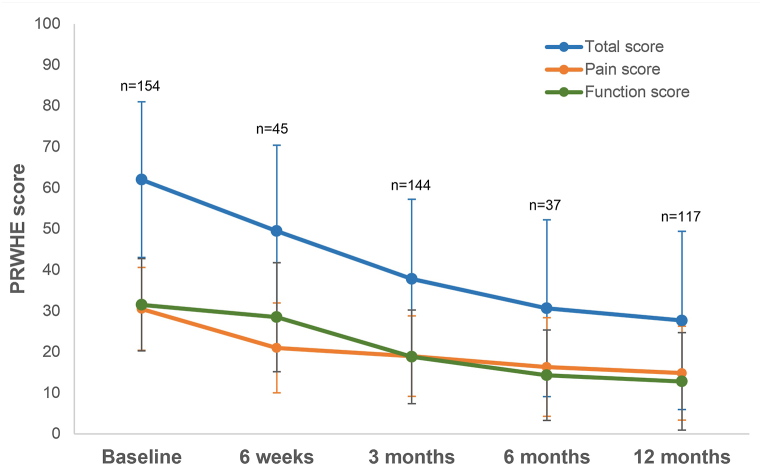
Patient-rated Wrist/Hand Evaluation (PRWHE) scores at baseline and 6 weeks, and 3, 6, and 12 months after surgery. Markers display mean scores; error bars display standard deviations.

The total PRWHE score improved significantly over time from 62 (SD: 19) at baseline to 28 (SD: 22) at 12 months postoperatively (P < 0.05). This improvement was attributed to significant changes in both pain and function scores (Fig. 4).

There were no differences in PRWHE scores between the groups with the greatest and least improvement in forearm rotation (first quartile: 38 [SD: 28] and fourth quartile: 38 [SD: 24], respectively).

Preoperatively, patients scored 49 (SD: 23) on the VAS scale for average pain. At 6 weeks after surgery, this pain score was significantly lowered to 25 (SD: 22) (p < 0.05). The score obtained at 12 months after surgery was 21 (SD: 22), indicating that the average pain did not improve further after the first 6 weeks.

Satisfaction with hand/wrist function improved significantly over time from 23 (SD: 19) at baseline to 73 (SD: 27) at 12 months postoperatively on the VAS scale (p < 0.05).

### 3.4 Radiographic evaluation

There was a wide range in preoperative radial inclination (−7°–37°) and volar tilt (−42°–29°). The distribution of preoperative tilt in our population was bimodal, which drove the mean closer to neutral. Most patients had a dorsal tilt, and a smaller part had a volar tilt. At baseline, 174 of the 175 patients had volar tilt or radial inclination outside the normal range (7–15° volar tilt, 21–25° radial inclination). The last patient was on the edge of both parameters. Four patients (2 %) had an articular step-off on the baseline radiograph.

Radial inclination, volar tilt, and ulnar variance differed significantly between baseline and follow-up at 3 months (Table 3) (p < 0.05).

**Table 3:** Radial inclination and ulnar variance on posteroanterior and volar tilt on lateral radiographs at baseline and 3 months after surgery.

	*Baseline n = 153*	3-month *follow-up n* = *157*	p-value
*Radial inclination (°),mean (SD)*	17 [8]	21 [6]	<0.001
*Volar tilt (°), mean (SD)*	−12 [14]	3 [9]	<0.001
*Volar tilt* < *0 (°) (n = 120)*	−17 [9]	2 [8]	<0.001
*Volar tilt* ≥ *0 (°) (n = 33)*	8 [9]	9 [9]	0.62
*Ulnar variance (−/0/+), n (%)*	12 [8]/52 (34)/89 (58)	23/45/32	<0.001

n: number of patients; SD: standard deviation.

A high degree of reliability was found for the radiographic measurements. The ICC for the radial inclination was 0.92 (95% CI: 0.84–0.97), and for the volar tilt, it was 0.87 (95% CI: 0.71–0.95).

### 3.5 Complications

There were 91 complications in 59 individual patients during the first year of follow-up which may be related to the surgery. Two patients (1%) had a superficial wound infection treated with oral antibiotics. A delayed union occurred in two patients (1%). Plate removal was performed in 39 patients (22%), and 19 patients (11%) underwent ulnar shortening osteotomy. In 29 patients (17%), another surgical intervention (e.g., trigger finger release, carpal tunnel release, or tenolysis) was performed.

## 4 Discussion

We studied the effect of corrective osteotomy for distal radius malunion on forearm rotation at 12 months after surgery. We found that the surgery resulted in a significant improvement in forearm rotation. Both the overall improvement of rotation and the pronation and supination separately were comparable with those reported by other studies [5,13,14,16,[24], [25], [26]]. Although patients achieved less forearm rotation compared with the contralateral side (94%), they had sufficient forearm rotation one year after surgery to perform their activities of daily living [10,11]. Fibrosis of both the pronator quadratus muscle and the volar joint capsule of the DRUJ after the fracture hematoma could contribute to this impaired supination. In general, patients experience greater inconvenience with restriction of supination rather than pronation, as it is more difficult to compensate for the former with shoulder movement [15]. The significant improvement in supination observed in this study may have contributed to the high satisfaction scores recorded.

The MCID of 19° improvement in forearm rotation was accomplished in 61.5% of the patients 12 months after surgery. This is a large proportion considering that 29% of our population had <19° limitation of forearm rotation at baseline. The indications for corrective osteotomy vary. This study investigated all patients with corrective osteotomy for malunion of the radius rather than only those with limitation of forearm rotation as an indication for corrective osteotomy. We chose to include all patients in the study to determine whether the surgery did not result in a decreased forearm rotation. When we divided baseline forearm rotation into quartiles, it became clear that patients with the worst baseline forearm rotation benefit most from surgery. This is a direct consequence of the greater potential for improvement. Although patients in the group without limitation of forearm rotation at baseline did not benefit from a corrective osteotomy in terms of improved forearm rotation, it was important that they did not worsen due to the surgery. Moreover, we found that the improvement in forearm rotation, especially supination, between baseline and 3 months continued between 3 and 12 months after surgery. The most significant improvement occurred in the first 3 months after surgery. The average 10° increase in the following 9 months may be within the measurement error and is of uncertain clinical relevance. On the other hand, this may show the combined effect of a malunion and soft tissue contractures as the cause of the impairment. Intensive and long-term training may have also contributed to improvement in rotation in our patients.

We found a significant increase in grip strength from 15 kg at baseline to 23 kg 12 months after surgery. However, there is still an appreciable reduction of about 15% in grip strength compared to the contralateral side. This was in line with the results of other studies [26]. Normative values of grip strength for females aged 50–65 years are 20.6–22.5 kg, corresponding to the postoperative mean grip strength measured 12 months after surgery in this investigation [27].

Our PRWHE score improved from 62 to 28 points postoperatively, without differences observed between the groups with the best and worst improvement in forearm rotation. This is a substantial and important improvement of 34 points, considering the MCID of 11.5 points reported in the literature [28]. Mulders et al. recorded a median postoperative PRWHE score of 18.5. Stirling et al. found a median PRWE score (of which the PRWHE score is the modified version) of 22. However, the median follow-up time of these studies was 27 months and 6 years, respectively, which may have resulted in better scores due to patients coping with their residual abilities [5,29]. From our results in combination with the results of the latter study, we can conclude that patients did not reach normal values (mean normative value of PRWE is 7.7), even in the long term [29,30].

In our study, the VAS score for average pain improved significantly from 49 at baseline to 21 at 12 months after surgery. In a study by Aibinder et al. pain improved from 58 to 20 points on the VAS scale. Although different preoperative pain scores were found, the postoperative pain was rated similarly to our study [13]. The VAS score of 73 for satisfaction with hand/wrist function at 12 months after surgery indicates that patients were satisfied with the functional outcome of the surgery. Improvement in forearm rotation may have contributed to these high scores, but possible improvements in flexion/extension, pain or cosmetic appearance also contribute to this.

The patients in our study had a wide variation in the direction and size of the distal radius malunion. The treating physician indicated the surgery in close consultation with the patient. This indication is mainly based on the symptoms experienced by the patient in combination with the presence of a malunion. It does not involve strict minimal radiological abnormalities, but a malunion was diagnosed when radiological parameters fell outside normal values. Most patients had a dorsal tilt (negative volar tilt) prior to surgery. However, some patients had an increased volar tilt. Both might be an indication for correction of the malunion if there was also pain or functional complaints. The primary purpose of corrective osteotomy is to align the distal radius and thereby improve the congruence of the DRUJ. The DRUJ is essential for forearm rotation. A restored DRUJ anatomy is therefore considered necessary to ensure the possibility of full forearm rotation. We measured radial inclination, volar tilt, and ulnar variance using preoperative and postoperative radiographs and detected significant changes in all three variables. A normal radial inclination (21–25°) was achieved in most patients. In our population, 68% of the patients had a negative or neutral ulnar variance postoperatively, while 11% secondarily underwent an ulnar shortening osteotomy for their remaining positive ulnar variance. A normal volar tilt (7–15°) was not achieved in our study (mean: 3°). This was in line with the findings reported by Madsen et al. who found a mean volar tilt of 4.5° (SD: 6°) after surgery with the same volar locking plates as those used in our study [31]. Stirling et al. found a mean postoperative volar tilt of 7°, but only 43% of their osteotomies were fixated with a volar locking plate. Filer et al. [32]. demonstrated that rotational deformity is common in displaced distal radius fractures. They found that 75% of the fractures had rotational angles greater than 5° in the longitudinal axis of the radius, which may influence the patient's ability to pronate or supinate. The rotational deformity can be difficult to measure on simple radiographs, but attempts were made to correct it visually during surgery. Our results show that a satisfactory long-term functional result can be achieved by restoring the radiological parameters as close as possible within normal values and using a volar locking plate.

This study has both strengths and limitations. We included 77 patients, while most sample sizes previously reported in the literature were smaller. In contrast to most other studies, we had both pre- and postoperatively collected data available [5,13,16]. Another strength was the generalizability of the results because of the large number of surgeons and the distribution of patients across the country. In addition, the inclusion of patients with all types of fractures (intra- and extra-articular) and primary treatment (conservative, operative) render the results of this study highly generalisable. The standardised post-surgery treatment supervised by a hand therapist was another strength of this study.

A limitation of our study is the substantial amount of missing data in our database. A significant number of patients are lost to follow-up after 3 months and could therefore not be included in our primary analysis. Although we do not have measurements of forearm rotation from the group that did not visit our clinic 12 months after surgery, we have no reason to assume that there is a selection of patients who did or did not return after 12 months. There is high heterogeneity in the study population in terms of malunion type, previous treatment, or amount and duration of symptoms. This is a result of including all patients that underwent a corrective osteotomy of distal radius malunion in our clinic. Since we know from the results of this study that patients with already good forearm rotation do not worsen as a result of surgery, we recommend including a more homogeneous population in a future study. There is a also large difference between patients in the timeframe between trauma and the corrective osteotomy (range 3–488 months). Some patients were symptomatic since their injury and were operated on relatively soon after the trauma (within a year). Therefore we can not completely rule out that the symptoms would also have improved by its natural course. However, our clinic does have a general practice that patients should first be treated conservatively to improve their symptoms. When symptoms do not improve conservatively, surgery is considered. Furthermore, our primary outcome was forearm rotation, which can be difficult to measure, particularly when assessed by less experienced clinicians [33]. We can also not completely rule out bias in the ROM measurements because hand therapists were not blinded. In our study, all measurements were performed by specialised hand therapists, following the same protocol in which they are extensively trained, thereby reducing the risk of measurement errors and bias. Nineteen patients (11%) developed such severe ulnar-sided wrist problems after the corrective osteotomy that an ulnar-shortening osteotomy was offered. It remains challenging to determine the correct ulnar variance based on two-dimensional radiographs. The use of three-dimensional planning and correction would possibly have improved the accuracy of the correction [25]. Thereby, it is unclear whether not performing this second surgery would also have led to improvement due to natural improvement.

## 5 Conclusion

In patients with reduced forearm rotation due to a distal radius malunion, a corrective osteotomy is an effective treatment which significantly improves forearm rotation. Patients with a symptomatic distal radius malunion without limitation of forearm rotation do not benefit from a corrective osteotomy in the improvement of forearm rotation; however, they also do not worsen as a result of the surgery. In addition, this intervention improves grip strength, the PRWHE-score, pain, and satisfaction even in patients without an improvement in ROM.

## Data availability statement

The data associated with this study will be made available on request.

## Funding

The authors received no financial support for the research, authorship, and/or publication of this article.

## Informed consent

Written informed consent was obtained from all subjects before the study.

## Ethical approval

The study protocol was approved by the Medical Ethical Committee of the Erasmus Medical Center (reference number: MEC-2018-1088).

## CRediT authorship contribution statement

**E.M. van Es:** Conceptualization, Formal analysis, Methodology, Project administration, Writing – original draft. **M. Dijkhof:** Methodology, Writing – original draft. **J.S. Souer:** Data curation, Methodology, Supervision, Writing – review & editing. **F.J. van Ewijk:** Data curation, Methodology, Writing – review & editing. **L. Hoogendam:** Data curation, Formal analysis, Methodology, Visualization, Writing – review & editing. **H.P. Slijper:** Conceptualization, Data curation, Resources, Software, Writing – review & editing. **R.W. Selles:** Conceptualization, Methodology, Supervision, Writing – review & editing. **J.W. Colaris:** Conceptualization, Supervision, Writing – review & editing.

## Declaration of competing interest

The authors declare that they have no known competing financial interests or personal relationships that could have appeared to influence the work reported in this paper.
